# 3D Printing of Functional Strawberry Snacks: Food Design, Texture, Antioxidant Bioactive Compounds, and Microbial Stability

**DOI:** 10.3390/antiox12020436

**Published:** 2023-02-10

**Authors:** Anica Bebek Markovinović, Predrag Putnik, Tomislav Bosiljkov, Deni Kostelac, Jadranka Frece, Ksenija Markov, Adrijana Žigolić, Jelena Kaurinović, Branimir Pavlić, Boris Duralija, Sandra Zavadlav, Danijela Bursać Kovačević

**Affiliations:** 1 Faculty of Food Technology and Biotechnology, University of Zagreb, Pierottijeva 6, 10000 Zagreb, Croatia; 2 Department of Food Technology, University North, Trg dr. Žarka Dolinara 1, 48000 Koprivnica, Croatia; 3 Faculty of Technology, University of Novi Sad, Blvd. cara Lazara 1, 21000 Novi Sad, Serbia; 4 Department of Pomology, Division of Horticulture and Landscape Architecture, Faculty of Agriculture, University of Zagreb, Svetošimunska Cesta 25, 10000 Zagreb, Croatia; 5 Department of Food Technology, Karlovac University of Applied Sciences, Trg J. J. Strossmayera 9, 47000 Karlovac, Croatia

**Keywords:** additive manufacturing, functional food, strawberry, starch, bioactive compounds, antioxidant capacity, rheology, microbiology

## Abstract

3D printing technology (3DP) as additive manufacturing is an innovative design technology that can meet the individual nutritional and sensory needs of consumers. Therefore, the aim of this work was to apply 3DP in the production of a strawberry-based functional product with the addition of two hydrocolloids (corn and wheat starch) in three proportions (10, 15 and 20%) and to investigate the influence of 3DP process parameters on physico-chemical and textural properties, as well as the bioactive and antioxidant potential and microbiological stability, with(out) the addition of natural antimicrobial agents. Starch type had a significant effect on all tested bioactive compounds, as well as on starch content, except for total phenolic and hydroxycinnamic acid contents. Considering the content of bioactive compounds and antioxidant capacity, program 2 proved to be more suitable than program 1. All samples exhibited good textural properties, a high degree of stability and minimal geometric deviations. Regarding microbiological safety, no pathogenic bacteria were found in the 3DP samples during storage. The 3DP sample with added citral at a concentration of 75 mg L^−1^ showed the best microbiological quality. Ultimately, 3DP can be successfully used for the production of new strawberry-based functional products.

## 1. Introduction

Consumers’ awareness between dietary habits and wellbeing is on a constant increase around the world, and it is particularly evident in the aftermath of the pandemic triggered by the SARS-CoV-2 virus. Research in 16 countries found that 42% of people will buy more functional foods in 2021 than in the previous year [1]. The increasing demand and consumption of functional foods is in line with guidelines for increased daily intake of fruits and vegetables with the aim of improving and maintaining health [2].

Fruits and vegetables are a rich source of various compounds, especially polyphenols, which have various beneficial effects. Due to its sensory properties (e.g., bright red color and pleasant aroma) and nutritional composition, strawberry is an attractive raw material for food manufacturing. Due to its beneficial effects on human health, strawberry is considered a functional food. This is contributed to by the bioactive compounds in the composition of strawberries, where the most important are polyphenols and their antioxidant properties, able to prevent the formation of free radicals. They are also involved in regulating the gene expressions responsible for metabolism, cell survival/proliferation, and DNA-repair mechanisms [3].

Despite all these benefits, current global consumption of fruits (e.g., strawberries) and vegetables remains insufficient. According to the recommendation of the WHO, at least 400 g of fruits and vegetables should be consumed daily to benefit from their intake [4]. Here, 3D food printing (3DP) represents a great potential for the development of interesting and nutritionally adapted food choices for consumers due to the versality of interesting shapes and designs (especially for children) [5].

The 3DP is a food manufacturing process that creates three-dimensional edible shapes (extrudates) by applying material(s) layer by layer. This approach offers a technological solution for the production of personalized foods with control over calorie content, final product composition, dimensions, and taste [6]. For example, Derossi et al. [7] designed a fruit-based meal for children 3–10 years old given that a snack must provide 5–10% of the total daily energy intake. Banana was the principal component in the meal because of its sensorial acceptance among children, plus it adds to the viscosity of the mixture. The rest of the ingredients fostered nutritional potential and consistency of the mixture for the printing. Severini et al. [8] also base their research on improving the nutritional content of the 3D printed form by preparing a balanced meal with fruits and vegetables. As mentioned earlier, fruits and vegetables are raw materials rich in beneficial compounds that can be used for 3DP functional manufacturing. Such products can be additionally enriched by adding other functional constituents, such as bioactive compounds (BAC), extracts from medicinal plants, algae, functional peptides, probiotics, etc.

However, one of the major challenges for 3DP food is microbial safety [9]. For this reason, additives are often inserted to 3D-printed mixtures to extend the shelf-life of the foods. The shelf-life of fruit-based 3D-printed products can be extended by adding natural food-grade antimicrobials such as vanillin [10,11,12], citral [11,13,14,15], and geraniol [12,16]. For instance, Cassani et al. [12] in their study observed a significant reduction of natural microflora (4–6 log cycles) in strawberry juice treated with vanillin and geraniol vs. control (untreated) samples. They also found a significant effect of vanillin on the increase of phenolic content of 3DP products. Another study showed that the combination of vanillin and citral with a mild heating can be an alternative to thermal processing of orange juice [15].

Another challenge with 3DP of fruit is that fruit is a raw material that cannot be easily printed due to its low soluble solids content; instead, it is necessary to add additives to the pulp that help in optimizing the viscosity and texture. These are primary substances that achieve a certain degree of thickening, such as hydrocolloid carriers. The most commonly used hydrocolloids in the food industry are pectin, xanthan gum, starch, carrageenan, guar gum, etc. Yang et al. [17] studied the rheological properties of 3D lemon juice gels prepared with different types of starches (potato, sweet potato, wheat, and corn) and concluded that the type of starch carrier modified the viscosity and mechanical properties in the final product. Azam et al. [18] studied the effects of different proportions of potato starch (15, 20, 25, and 30%) in orange concentrate on the rheological properties of the final product and concluded that the addition of 20% starch carrier gave a product with the best mastication properties. Liu et al. [19] conducted a similar study adding 0, 1, 2, and 4% potato starch to a mixture of mashed potato and 15% trehalose and concluded that the addition of 2% starch gave a product with the best extrudability and printability. Mu et al. [20] concluded that the addition of different proportions of berry powders (5, 10, and 15%) to potato starch paste significantly increased the content of total phenols and antioxidant activity in the final product. Thus, to produce 3DP products with good nutritional and biological value, as well as satisfactory rheological and textural properties, it is necessary to choose a suitable carrier and optimize its ratios.

From the abovementioned, the derived aim of this work was to apply 3D printing technology in the production of a functional strawberry products using wheat and corn starches in three different designs/proportions of 10%, 15%, and 20% (*w*/*w*) and to study the physico-chemical and rheological properties of the final products. The bioactive compounds and antioxidant capacity were monitored before and after the 3DP to evaluate the bioactive compounds’ functionality. Since 3DP products have a very short shelf-life, a 10-day storage at 4 °C was tested with natural antimicrobials (citral and vanillin) for extending microbiological stability.

## 2. Materials and Methods

### 2.1. Chemical and Standards

HPLC 99% pure methanol obtained from Honeywell (Paris, France) was used as an extraction solvent, while the Folin–Ciocalteu reagent obtained from Fisher Scientific UK (Loughborough, UK) was used for spectrophotometric determination of total phenols. Hydrochloric acid (37%, *w*/*w*), sulfuric acid (96%, p.a.), sodium carbonate, anhydrous (99.5–100.5%), and formic acid (98%, p.a.) were obtained from Lach-ner (Neratovice, Czech Republic). Ethanol (96% pure) was obtained from Gram-mol (Zagreb, Croatia). Quercetin (95%), potassium acetate (99%), and aluminum chloride (98.5%) were purchased from Acros Organics (Guangzhou, China). Gallic acid standard (97.5–102.5%), DPPH (2,2-diphenyl-1-picrylhydrazyl radical), and TPTZ (2,4,6-tris-2-pyridyl-s-triazine) were obtained from Sigma-Aldrich (St. Louis, MO, USA). Vanillin (99%), potassium chloride (99.0–100.5%), sodium acetate anhydride (99%), and chlorogenic acid (min. 95%) were purchased from Thermo Fisher (Kandel, Germany). Iron (III)-chloride hexahydrate and sodium acetate trihydrate resistant to potassium permanganate were obtained from Kemika (Zagreb, Croatia). Glacial acetic acid (≥99.8%) was purchased from Honeywell Fluka ^TM^ (Seelze, Germany) and trolox standard (6-hydroxy-2,5,7,8-tetramethylchroman-2-carboxylic acid) used for the DPPH and FRAP method was purchased from Biosynth (Bratislava, Slovakia).

### 2.2. Preparation of Fruit Material

The basic raw material for the 3D printing mixture(s) was strawberry juice blends (*Fragaria x ananassa* Duch., cv. ‘Albion’). The strawberries were purchased from Jagodar-HB Ltd. in Donja Lomnica, Zagreb County (Croatia). After the strawberries were delivered to the laboratory, they were destemmed, washed, dried with cellulose, packed in plastic bags, and stored at −18 °C. The strawberries were crushed and homogenized with a household blender to obtain a strawberry juice blend. The pure juice blends were used for the control samples, while starch was added to the other samples according to the experimental design (Table 1). Corn starch (Gustin Dr. Oetker, Janossomorja, Hungary) and wheat starch (Denes Natura Kft., Pécs, Hungary) were used in proportions of 10%, 15%, and 20% (*w*/*w*). The mixtures of strawberry pulp and various starches were continuously mixed and heated on a magnetic stirrer LLG-uniSTIRRER 7 (Lab w Group GmbH, Meckenheim, Germany) to a constant temperature (about 70 °C) to increase the viscosity and homogeneity. After heating, the mixtures were cooled down to a room temperature and subjected to 3D printing.

### 2.3. 3D Printing of Snacks

Foodini 3D printer (Natural Machines, Barcelona, Spain) was used to 3D print the mixtures. Capsules with a volume of 100 mL and a nozzle diameter of 4 mm were used and filled with mixtures of strawberry pulp and starches at room temperature. The filled capsules were inserted into a holder inside the 3D printer. The selection of the desired shape and other printing parameters were set using the Foodini Creator Software on the computer. A heart shape with three layers was selected for 3D printing. The 3D printing was performed with two different programs that differed in process parameters. Program 1 was set up with a printing speed of 8000 mm min^−1^, a printing line thickness of 3.5 mm, a mixture flow rate of 1.4, and a nozzle height of the first layer of 6 mm. Program 2 was set up: Printing speed 14,000 mm min^−1^, print line thickness 3.4 mm, mixture flow rate 1.65, and nozzle height of the first layer 4.5 mm. 3D printing was performed at room temperature. A mixture of strawberry pulp with the addition of wheat or corn starch in different percentages was printed by both programs (Table 1). Subsequently, pH and water activity were measured while extracts were prepared from samples for further analysis for determination of bioactive compounds and antioxidant capacities.

### 2.4. Characterization of 3DP Products

#### 2.4.1. Determination of pH and Water Activity (a_w_)

The pH was determined with a previously calibrated pH meter (Mettler-Toledo GmbH, Greifensee, Switzerland), by immersing the combined electrode into a homogenized sample previously diluted with distilled water in a 1:1 ratio and reading the pH. The read results were expressed as the mean of two parallel measurements ± standard deviation.

Water activity was determined using an a_w_ meter (AquaLab, P08584, Decagon Devices, Pullman, WA, USA). The instrument was calibrated before the first use. For measurement, the sample was applied to a standard measuring cup so that the entire surface of the cup was covered with the sample. The results were expressed as the average of two parallel measurements ± standard deviation.

#### 2.4.2. Determination of Bioactive Compounds and Antioxidant Capacity

Extraction of bioactive compounds was performed on an ultrasonic processor (UP400St, Hielscher Ultrasound Technology, Teltow, Germany) coupled with titanium DN22 (546 mm^2^) sonotrode according to a modified protocol from the literature [21]. An aqueous solution of methanol (80%, *v*/*v*) with 1% formic acid (*v*/*v*) was used as the extraction solvent. Ten grams of the sample was placed in a beaker and 40 mL of the extraction solvent was added. Then, ultrasound-assisted extraction was performed under the following conditions: Amplitude 50%, pulse 100%, and extraction time 5 min. After extraction, the extract was filtered into a 50 mL volumetric flask and made up with the extraction solvent. The obtained extracts were stored at 4 °C until the analysis. The prepared extracts were used for all determinations of bioactive compounds and antioxidant capacity with a spectrophotometer (LLG-uniSpec 2, Spectrophotometer, Meckenheim, Germany) in parallel determinations.

- Determination of Total Phenolic Content (TPC)

A modified Follin–Ciocalteu method from the literature was used to determine the TPC content [22] and the determination procedure is identical to previous work by Bebek Markovinović et al. [23]. The TPC was calculated using a calibration curve prepared with different concentrations of gallic acid solutions (10–250 mg L^−1^), and the results were expressed as mg gallic acid equivalent (GAE) per 100 g of sample.

- Determination of Total Monomeric Anthocyanins (ANT)

Anthocyanins were determined by the spectrophotometric differential pH method [24], the determination procedure being identical to previous work by Bebek Markovinović et al. [23]. The concentration of monomeric anthocyanins in the sample was expressed as pelargonidin-3-glucoside equivalent (Pg-3-G) (mg 100 mL^−1^).

- Determination of Total Flavonoids (TF)

A modified spectrophotometric method described in the literature [25] was used for the determination of TF. Briefly, 0.5 mL of the extract, 1.5 mL of 96% ethanol, 0.1 mL of 10% aluminum chloride, 0.1 mL of 1 M potassium acetate, and 2.8 mL of distilled water were pipetted sequentially into a test tube. The blank sample was prepared in the same way, but instead of the extract, an extraction solvent was taken, and instead of 10% aluminum chloride, distilled water was added. The reaction mixture was briefly homogenized on a vortex device, and after 30 min, the absorbance was measured at a wavelength of 415 nm. Parallel measurements were performed for each sample. A quercetin standard solution (10–200 mg L^−1^) was used to construct the calibration curve, and results were expressed as mg quercetin equivalent (QE) per 100 g of sample.

- Determination of Total Hydroxycinnamic Acids (HCA) and Total Flavonols (FL)

HCA and FL were determined using a modified spectrophotometric method [26] and the determination procedure is identical to previous work by Bebek Markovinović et al. [23]. The HCA content was calculated using a calibration curve generated with different concentrations of chlorogenic acid (10–600 mg L^−1^), and the results were expressed as mg chlorogenic acid equivalent (CAE) per 100 g or 100 mL of the sample. The FL in the extracts was calculated from a calibration curve obtained with different concentrations of quercetin solution (10–600 mg L^−1^) and the results were expressed as mg quercetin equivalent (QE) per 100 g of the sample.

- Determination of Condensed Tannins (CT)

CT was determined by a modified spectrophotometric method [27] and the procedure for determination is identical to previous work by Bebek Markovinović et al. [23]. The CT in the extracts were calculated from a calibration curve obtained with different concentrations of catechin solution (10–120 mg L^−1^), and the results were expressed as mg catechin equivalent (CA) per 100 g of the sample.

- Determination of Antioxidant Capacity (AOC)
  1. DPPH Method
    The antioxidant activity was determined using the spectrophotometric DPPH method described in the literature [28]. Briefly, 1.5 mL of the properly diluted extract and 3 mL of a 0.5 mM DPPH solution were pipetted into a glass test tube. As a control, 1.5 mL of 100% methanol and 3 mL of 0.5 mM DPPH solution were pipetted. Pure methanol was used as a blank. After setting up the reaction, the test tubes were kept in the dark for 20 min and then the absorbance was measured at 517 nm. Parallel measurements were performed for each sample. Antioxidant activity was calculated using a calibration curve generated with different concentrations of Trolox solution (10–150 µM) and the results were expressed as µM rolox equivalent (TE) per 100 g of the sample.
  2. FRAP (Ferric Reducing Antioxidant Power) Method
    Another method used to determine antioxidant activity was the spectrophotometric FRAP method described in the literature [29]. Briefly, 600 μL of the previously appropriately diluted extract and 4.5 mL of the FRAP reagent (prepared from acetate buffer (0.3 M), 2.5 mL of TPTZ reagent (2,4,6-tris-2-pyridyl-s-triazine; 10 mM), and 2.5 mL of iron (III) chloride (20 mM) in a ratio of 10:1:1) were pipetted into the glass tubes. Briefly, the reaction mixture was homogenized on a vortex shaker (Grant Instruments Ltd., Cambs, England) and thermostatted in a water bath at 37 °C for 10 min. For the blank sample, the determination procedure was identical except that the extraction solvent was used instead of the extract. After 10 min, the absorbance was read at 593 nm. Ferric reducing antioxidant power was calculated from a calibration curve obtained with different concentrations of Trolox solution (10–150 µM), and the results were expressed as mM Trolox equivalent (TE) per 100 g of the sample.

### 2.5. Determination of Color Parameters

Color analysis was performed using a Lovibond colorimeter (LC 100, Lovibond—Tintometer Ltd., Amesbury, UK). Prior to analysis, the colorimeter was calibrated with an integrated white reference slider. Color was expressed as L*, a*, and b* values where L* indicates lightness, a* redness, and b* yellowness. All readings were taken at five random locations from the surface of each sample and the average was used to express the color of the individual sample.

Color difference (ΔE*), chroma (C*), and hue angle (*h*) were calculated using Equations (1)–(3) [30,31]:

(1) $$\Delta E^{*}=\sqrt{{\left(\bar{L^{*}}-\bar{L_{0}^{*}}\right)}^{2}+{\left(\bar{a^{*}}-\bar{a_{0}^{*}}\right)}^{2}+{\left(\bar{b^{*}}-\bar{b_{0}^{*}}\right)}^{2}}$$

(2) $$C^{*}=\sqrt{{\left(\bar{a^{*}}\right)}^{2}+{\left(\bar{b^{*}}\right)}^{2}}$$

(3) $$h\;{}^{\circ}={\mathrm{tg}}^{-1}\left(\frac{\bar{b^{*}}}{\bar{a^{*}}}\right)$$

where $\bar{L^{*}}$, $\bar{a^{*},}\;\bar{b^{*}}\;$are the mean values of the measured L*, a*, b* parameters of the 3D-printed samples; and $\bar{L_{0}^{*}},\;\bar{a_{0}^{*}},\;\bar{\;b_{0}^{*}}$ are mean values of the measured L*, a*, b* parameters of the control sample.

### 2.6. Texture Analysis

Texture properties were determined using a TA.HD.plus Texture Analyser (Stable Micro System, Godalming, UK). With different extensions, two tests were performed: a penetration test and an extrusion test.

#### 2.6.1. Penetration Test

Tests were performed using a spherical probe of 4 mm. Texture analyzer settings were set to: pre-test speed, 1 mm s^−1^; test speed 0.5 mm s^−1^; post-test speed 10 mm s^−1^; deformation distance 6 mm; and trigger force 2 g. The measurements were carried out at room temperature. Mean values in triplicates were reported as the maximum force (F_p_) (hardness) and work (W_p_). The sample strength was presented as the breaking force (N) concerning deformation distance (mm).

#### 2.6.2. Forward Extrusion Test

Tests were conducted with an extrusion cell (sample container and the piston disc). The sample container can settle the base disc with an outlet diameter of 3 mm. Selected diameters were chosen depending on the consistency of the sample. The parameters were as follows: pre-test speed 1 mm s^−1^; test speed 1 mm s^−1^; post-test speed 10 mm s^−1^; deformation distance 20 mm; and trigger force 10 g (0.098 N). A compression force–time curve was obtained. The measurements were carried out at room temperature, results were calculated from triplicated observations and shown as the mean extrusion force (F) (firmness) and work (W).

### 2.7. Microscopic Analysis

The microstructures of the printed samples were evaluated using a digital microscope with an adjustable maximum magnification of 500× (Dino-Lite Edge Digital Microscope AM 7915 MZT, Dino-Lite Europe/IDCP B.V., Almere, The Netherlands). Images were captured and recorded using Dino Capture 2.0 software (Dino-Lite Europe/IDCP B.V., Almere, The Netherlands).

### 2.8. Particle Size Distribution and Dimension Measurement of 3DP Samples

The particle size distribution was measured using laser diffraction analysis (Malvern Mastersizer 2000, Malvern Instruments Ltd., Worcestershire, UK) with the liquid dispersion system Hydro 2000S. Approximately 10 g of each sample was dispersed in 30 mL of distilled water to obtain a homogeneous solution adequate for measuring at the minimum achieved degree of obscuration. The particle diameters were expressed over: d (0.1)—10% of the volume distribution is below the observed diameter; d (0.5)—median diameter, 50% of the volume distribution is below, and 50% is above the observed diameter; d (0.9)—90% of the volume distribution is below the observed diameter; D (3.2)—surface weighted mean diameter (Sauter mean diameter); D (4.3)—volume weighted mean diameter (De Brouckere mean diameter).

Dimensions of 3DP samples were measured over three geometric values (length × width × height). Changes in a variety of dimensions were detected using a digital caliper with an accuracy of 0.01 mm. The default dimensions of the 3DP product according to both programs were: 53 mm × 51 mm × 12 mm (length × width × height).

### 2.9. Shelf-Life Study

The microbiological quality of 3DP samples was monitored during storage at 4 °C for 10 days. A total of 5 samples were analyzed as follows: (i) control sample—without antimicrobial agents; (ii) 3DP sample with the addition of vanillin 1 g L^−1^; (iii) 3DP sample with the addition of vanillin 2 g L^−1^; (iv) 3DP sample with the addition of citral 75 mg L^−1^; and (v) 3DP sample with the addition of citral 150 mg L^−1^. The selected antimicrobial concentrations were adopted and modified according to previous findings [15,32].

Classical microbiological methods were employed, and microorganisms selected for the analysis were in accordance with the prescribed regulations for foodstuffs (EC, 2073/2005) [33]. Here, 1 g of a 3D-printed sample was homogenized in 9 mL of sterile deionized water and serial dilutions were made before plating. For the determination of total aerobic mesophilic bacteria (AMB), the pour plate method was used and for other bacteria, the spread plate method was employed on appropriate selective media. AMB count was determined after incubation on Nutrient agar (Merck, Darmstadt, Germany) at 37 °C for 24–48 h. Molds and yeasts were determined after incubation on Potato Dextrose agar (Biolife, Milan, Italy) at 25 °C for 96 h. Enumeration of *Enterobacteriaceae* was conducted on Violet Red Bile Glucose (VRBG) agar (Biolife, Milan, Italy) after incubation at 37 °C for 48 h. *Salmonella* sp. detection was conducted in a Rappaport–Vassiliadis (RV) Salmonella enrichment broth (Merck, Darmstadt, Germany), and by subculturing on Xylose Lysine Deoxycholate (XLD) agar (Biolife, Milan, Italy) at 37 °C for 24–48 h. All experiments were conducted in triplicate and the results were expressed as the colony forming units per gram of 3D-printed sample (CFU g^−1^).

### 2.10. Statistical Analysis

Descriptive statistics were used to characterize pH, a_w_, bioactive compounds, and antioxidant capacities of the 3DP samples. Multivariate analysis of variance (MANOVA) simultaneously tested associations among dependent and independent variables. The significance level for all tests was α ≤ 0.05, and the results were analyzed using SPSS software (v.22) (IBM, Chicago, IL, USA). Statistical analysis of texture analysis, particle size distribution and color parameters were performed using “Statistica 12” software (TIBCO, Palo Alto, CA, USA).

## 3. Results and Discussion

### 3.1. The Influence of 3DP Technology on a_w_ and pH

Considering that fruit is a raw material with a low pH but also with a high water-activity, in this study, the influence of the process parameters of the 3DP technology on the stability of these parameters was monitored (Table 2). The average water activity in the 3D-printed samples was 0.95%. In contrast to the program type, which had no statistically significant effect on the a_w_ value, the starch type and starch content did have a significant effect on this variable. Here, samples with wheat starch had a higher a_w_ value than samples with corn starch. The results of Dogan et al. [34] showed that among the starches tested, such as modified corn starch, unmodified corn starch, modified potato starch, tapioca starch, potato starch, and wheat starch, corn starch had the best swelling capacity. Our results could be explained by the fact that corn starch has a better swelling power and binds to water better than wheat starch (which reduced the a_w_ values in the samples). Increasing the starch content also resulted in an increase in the a_w_ value compared to the initial value at 10% starch content. This could be due to the fact that glucose and sucrose, which are naturally present in strawberries, compete for water binding [35,36] and are inversely related to the a_w_ value. Thus, as starch content increases, the concentration of these sugars in the samples decreases, which may be reflected in an increase in a_w_ values.

The average pH of 3D-printed samples was 3.53. All three variables studied, starch type, starch content, and 3D program, had a statistically significant effect on the pH of the samples. Higher pH values were found for the 3D printed product with wheat starch at higher proportions of added starch (15% and 20% vs. 10%) printed with program 2. These results agree with the findings of Rababah et al. [37], who found a significant effect of the addition of hydrocolloids on pH in gelatinized strawberry products. Moreover, Bravo-Núñez et al. [38] indicated that the interaction between starch and proteins could affect pH. Therefore, it is possible that studied starches bind differently to strawberry proteins and thus affect the pH of the 3DP product.

### 3.2. The Influence of 3DP Technology on the Stability of Bioactive Compounds and Antioxidant Capacity

The results for the effect of 3DP processing parameters on the bioactive compounds content of and antioxidant capacity in the 3DP samples are shown in Table 3.

The mean value of total phenols in the 3DP samples was 78.57 ± 1.08 mg 100 g^−1^ sample. Of all analyzed bioactive components, condensed tannins were the most abundant (44.46 ± 0.19 mg 100 g^−1^), followed by hydroxycinnamic acids (16.54 ± 0.18 mg 100 g^−1^), anthocyanins (7.56 ± 0.02 mg 100 g^−1^), flavanols (5.30 ± 0.12 mg 100 g^−1^), and finally, total flavonoids (4.90 ± 0.04 mg 100 g^−1^).

The type of starch carrier had a statistically significant effect on all bioactive compounds tested. The content of total phenols, anthocyanins, and condensed tannins was higher in 3D-printed samples with wheat starch, while the content of hydroxycinnamic acids, flavanols, and total flavonoids was higher in samples with corn starch.

Starch carrier content affected (*p* ≤ 0.05) contents of all bioactive compounds except those of total phenols and hydroxycinnamic acids. Increasing the starch content increased almost all dependent variables, up to 15% of the content. After the addition of 15% starch, the experimental mean values tended to either decrease or remain the same. Increasing the content of starch carriers (from 15% to 20%) in mixtures for 3D printing leads to a decrease in the mass fraction of all studied bioactive compounds. This result is expected considering that mixtures with a higher content of starch carriers have a lower content of the fruit component, which is the source of the bioactive compounds. Moreover, these results indicated that the addition of starch at the level of 15% is optimal for the preservation of bioactivity of the samples.

The type of 3D program had a statistically significant effect on the content of almost all bioactive compounds, except for hydroxycinnamic acids and flavonols, which were not affected. Program 2 compared to Program 1 increased the content of TPC (5%), TF (5%), ANT (4%), and CT (2%). Considering that the printing programs differed from each other in terms of the speed of nozzle movement, the speed of extrusion of the mixture from the nozzle, the thickness of the printing line, and the distance of the nozzle from the surface when printing the first layer, it is possible that due to the change in the different printing parameters, there was a faster degradation of TPC, TF, ANT, and CT with program 1, and consequently, their content differed according to the 3D-printing program.

The antioxidant activity of the 3DP samples was determined using two in vitro assays. The average values for DPPH and FRAP were 486.96 ± 0.29 µM and 1.24 ± 0.01 mM, respectively (Table 3). Starch type affected antioxidant capacity, with higher DPPH values detected in 3DP samples with corn starch and higher FRAP values in 3DP samples with wheat starch. Considering the starch content, the FRAP values followed the trend of the content of other bioactive compounds, i.e., with increasing starch content, the FRAP value increased, but mostly only up to a proportion of 15%, after which it tended to decrease with further increasing starch content. An exception was DPPH, whose value decreased with increasing starch content (−12%). These results are consistent with a similarly designed study by Mu et al. [20]. In their study, potato starch pastes were fortified with 5, 10, and 15% strawberry or blackcurrant powders, and significantly higher DPPH and FRAP levels were found in such pastes compared to pure starch paste. In other words, DPPH and FRAP values correlated with the content of total phenols from berry powders [20]. In our case, there was an expected decrease in the value of DPPH antioxidant capacity, since with an increase in the proportion of starch carriers, the proportion of strawberry pulp rich in bioactive compounds decreased. Regarding the type of 3D program, FRAP was not changed when a different programming of the 3D printer was selected. On the other hand, compared to Program 1, Program 2 increased the DPPH value by 0.7% and the FRAP value by 4%. These results somehow suggest that program 2 is more suitable for 3D printing strawberry-starch products, not only because of the higher antioxidant capacities, but also because of the higher levels of other biologically active compounds compared to Program 1.

Overall, for the production of functional foods, it is of particular importance to choose raw materials with a favorable bioactive compositions [39]. However, during processing, the quality of the raw material may be compromised and the final product may fall short of the starting raw material in terms of bioactive potential and antioxidant capacity. In this regard, 3DP technology with the principle of cold extrusion promises production of functional foods in which the deterioration of the quality of the original raw material would be extremely low [40]. However, the addition of various additives may affect the quality, which remains to be investigated.

### 3.3. The Influence of 3DP Technology on Color Parameters, Microscopic Analysis, and 3DP Dimension Measurement

Color is a key attribute that determines consumers’ acceptance of a food product and influences their purchasing behavior [30,31]. Table 4 shows the CIEL*a*b* color parameters in respect to variations in 3DP program, starch type and starch content. As can be seen, with no influence of 3DP operating conditions, ΔE* values greater than 12 represent a large color difference that can be easily perceived by human eyes [41].

Significant influence of 3DP operating conditions on colorimetric parameters were given in Table 5. The changes in lightness (L*) were influenced by the proportion of starch added, while the type of starch and type of printing program did not vary (Table 5).

**Figure 1:**
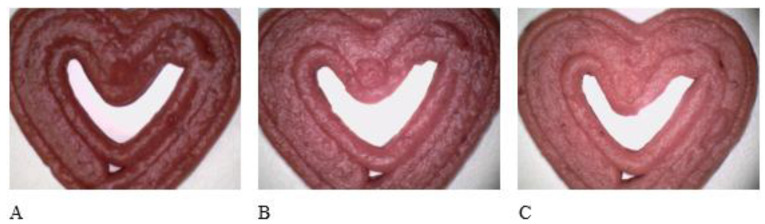
Microscopic pictures (500×) of 3DP samples: Influence of added starch, 10% (**A**); 15% (**B**); 20% (**C**), on the lightness (L*).

Increasing the proportion of the two added starches (corn and wheat) from 0 to 20% increased the lightness of samples (Table 4). Figure 1 shows photographs of printed strawberry samples with 10% (A), 15% (B), and 20% (C) starch content, confirming the information given previously. This was aligned with previous results about content of polyphenols, and antioxidant activity, as some of them are main carries of the color in the strawberry pulp (e.g., anthocyanins). So, it makes sense that samples were lighter with an addition of starch.

As shown in Table 5, the 3DP processing parameters did not significantly affect changes in the dimension of the 3D-printed product. Considering the results of mechanical properties, it was expected that the variation in starch type and starch content affected the variation in observed product dimensions. All samples show a high level of stability and minimum deviations in geometry (Table 6). This is very important for larger food manufacturing and the associations among food design in terms of a content and its geometric form (i.e., regardless of the added ingredient shape remains unchanged).

The samples containing 10% starch were visually much darker than the samples with 20% starch. A similar phenomenon was reported by Liu et al. [42], who investigated the effect of soluble starch-based additives on the color of 3D-printed beef, and found that the incorporation of starch-based additives gave the samples a lighter color. Zhang et al. [43] also found that the addition of starch increased the brightness of surimi beef gels. Furthermore, changing the printing programs, starch types (corn and wheat), and starch content did not result in significant changes in a*, b*, C*, and h (*p* > 0.05). All printed samples contained approximately equal amounts of a*, b*, C*, and h despite the different printing programs, starch types, and starch contents, and the only difference between samples was brightness.

### 3.4. The Influence of 3DP Technology on Texture Properties and Particle Size Distribution

Changing the proportion of the two added starches resulted in a significant change in the work (W_p_) and force (F_p_) required to penetrate the samples, as shown in Table 7.

Force (F_p_) and Work (W_p_) increased with increasing starch content, implying sturdier (firmer) texture in the samples. A similar trend was reported by Yang et al. [44], who studied the effect of potato starch (10, 12.5, 15, 17.5, and 20 g 100 g^−1^) on the textural properties of lemon juice gels. In addition, Feng et al. [45] added potato starch (2, 4, 6, and 8%) to improve the printability of Nostoc sphaeroides gel, while Dong et al. [46] examined the addition of starch (2, 6, 8, and 10%) to surimi-based printing mixtures. In all cases, with the increase of starch content in the gels, their hardness also increased and they were more resistant to external damage. It is assumed that the increase in starch content leads to a greater number of starch molecules per unit volume, thus increasing the probability of intermolecular hydrogen bonding, which leads to greater compactness of the network structure and thus, to the strength of the samples [17,46]. In addition, the F_p_ values were also affected by added types of starch (Table 5). Here, samples containing corn starch exhibited significantly higher penetration force than those containing wheat starch.

We showed that the force and work required for the extrusion of mixtures significantly depend on starch types and its contents. Similar to the previously described results of the penetration test, the values for F and W increased with increasing starch content. Although increasing both starch types increased F and W values, the extent of the increase was greater for the wheat vs. the corn. This was in contrast to the results of the penetration test, where samples with corn starch had higher hardness than samples with wheat. Zheng et al. [47] found that wheat starch forms stronger starch gels than corn starch when performing texture analysis on 3D-printed samples of wheat and corn starch. This suggests that the reason for the difference in gel strength may be the higher amylose content of wheat starch compared to corn starch. It can be assumed that the extrusion test still describes the texture of the observed samples more reliably than a penetration test. Measurement errors in the case of a penetration test are possible due to the specific geometry of the 3D-printed samples and the flat-bottomed cylindrical probe chosen as an extension for the texture analyzer. Considering that the surface of the measured samples was not completely flat and smooth, the use of spherical probes instead of cylindrical probes could increase the precision of the mentioned method.

In addition, textural properties are also affected by printing parameters such as nozzle diameter, nozzle height, extrusion speed, printing speed, and line thickness [48,49], which was not the case here. Namely, the selected printing programs (programs 1 and 2) did not significantly affect the texture of the samples, although they differed from each other in the first layer nozzle height, print speed, line thickness, and extrusion speed. It is assumed that this is not due to insufficient deviations in the values of the specified parameters according to which the programs differed.

The variations in starch concentration resulted in significant changes in the values of d (0.1), d (0.5), d (0.9), D (3.2), and D (4.3). As shown in Table 8, an increase in starch content resulted in a decrease in the observed particle size distribution parameters. By decreasing the values of d (0.1), d (0.5), d (0.9), D (3.2), and D (4.3), the increase in smaller particles becomes more evident. In addition, the change of d (0.1), D (3.2), and D (4.3) was also significantly affected by the starch type (*p* ≤ 0.05). As shown in Table 8, the samples containing corn and wheat starch at the same concentration had lower particle size distribution values than the samples with wheat starch added.

**Figure 2:**
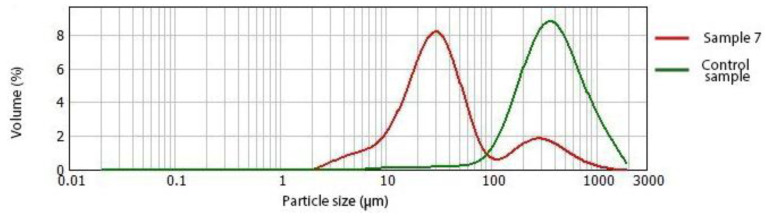
Volume size distribution of control sample (green), and sample 7 with 20% of corn starch (red).

The volume size distribution (Figure 2) shows that the addition of 20% starch increased interval particles of size 2–85 μm compared to the reference sample without starch and a decrease in particle size between 85 and 1673 μm.

By comparing the particle size distribution results with the extrusion test results, it was observed that samples with smaller particles were more difficult to extrude than samples with larger particle sizes. The correlation of the particle size distribution with the textural properties of the printed samples was also observed in the results of the penetration test, where the strength of the printed samples increased as the proportion of smaller particles increased. Wilms et al. [50] demonstrated a correlation between individual material properties, e.g., particle size, and the extrusion process. According to the previously mentioned studies, the Consistency factor (K) is inversely proportional to the particle size. On the other hand, the consistency factor affects the apparent viscosity and thus, the pressure required for the extrusion process, which increases when the K value is increased. This explains the increase in the maximum force and work required to extrude strawberry samples when the particle size is decreased. In addition, the reduction in particle size can be used to improve processability [50].

### 3.5. Microbial Analyses of 3DP Samples

Microbial analysis of 3DP samples was conducted during the 10 days of storage at 4 °C. Results are presented in Table 9. Results indicated that none of the samples contained pathogenic bacteria or yeasts and molds, even after 10 days of storage. Total bacterial count was above the desired limit for the samples 2 and 4.

The samples with added vanillin (1 g L^−1^) were stable during the 4-day storage period, after which an increase in bacterial counts above the desired level was observed on days 7 and 10. Interestingly, in the samples with higher vanillin concentration (1 g L^−1^), a high bacterial count was observed only on one day (4th day), after which the count was within the desired range. These results leave open the possibility of inadequate homogenization of the small amounts of the added microbial agent in the strawberry matrix, which would result in a localized presence of the inhibitory agent, while there could be areas of decreased antimicrobial activity. In addition, although the sampling of the products was done in triplicate and different parts of the product were analyzed, there could be areas of the product that are susceptible to faster spoilage because the conditions in the external and internal parts of the product are very different. In addition, a reduction in aerobic mesophilic bacteria was observed at the citral concentrations studied during the storage. The inhibitory effect of citral could play a role in inhibiting bacterial growth during storage. The presence of aerobic mesophilic bacteria observed before storage could be due to handling during the preparation of the product and is limited to the surface. In addition, the problem of homogenization of the antimicrobial agent in the sample could lead to different citral concentrations in the sample. Further studies should address the technical problem of delivery of antimicrobial agents in products of this type. From our results, it is clear that the product formation process is free from pathogenic contamination and that the addition of citral (75 mg L^−1^) gave the best microbiological quality. However, the manufacturing methods need to be articulated to avoid localized antimicrobial activity in the final products.

## 4. Conclusions

The production of strawberry-based functional snacks with 3D printing technology had favorable effects on the retention and stability of the bioactive components and antioxidant capacity. The type of starch significantly affected the stability of all determined bioactive compounds and antioxidant capacity. The use of corn starch resulted in a higher level of hydroxycinnamic acids, flavanols, total flavonoids, and antioxidant capacity as determined by the DPPH method, while the product with wheat starch had higher levels of total phenols, anthocyanins, condensed tannins, and antioxidant capacity determined by the FRAP method. Starch content has a significant effect on almost all of the determined bioactive compounds and antioxidant capacity. To maintain the bioactive profile of the 3D-printed product and strong antioxidant capacity, a starch content of 15% is most favorable. The type of 3D printing program has a significant effect on the majority of the determined bioactive compounds and antioxidant capacity, where the use of program 2 resulted in greater stability of all analyzed components in the strawberry-based 3D printed product.

In terms of textural properties, the extrusion test better describes the mechanical properties of smaller particles affecting the strength and stability of 3DP products in relation to the applied 3DP processing parameters when both test mechanisms are used.

The microbiological safety of the product was confirmed as no pathogenic bacteria were detected in the samples during 10 days of storage at 4 °C. Further research should be directed towards the availability of antimicrobial agents in the strawberry matrices for 3D printing.

In conclusion, 3D printing can be considered a very promising technology with great potential for the development of innovative and customized functional products.

## Figures and Tables

**Table 1:** Design of the experiment.

Sample	Starch Type	Starch Content	3D Program
1	Control sample	-	-
2	Corn starch	10%	Program 1
3	Corn starch	10%	Program 2
4	Corn starch	15%	Program 1
5	Corn starch	15%	Program 2
6	Corn starch	20%	Program 1
7	Corn starch	20%	Program 2
8	Wheat starch	10%	Program 1
9	Wheat starch	10%	Program 2
10	Wheat starch	15%	Program 1
11	Wheat starch	15%	Program 2
12	Wheat starch	20%	Program 1
13	Wheat starch	20%	Program 2

Sample 1—Control sample without added starch.

**Table 2:** Association of 3DP parameters with the contents of a_w_ and pH in the 3DP samples.

Variable	*n*	a_w_	pH
Starch type		*p* ≤ 0.01 ^†^	*p* ≤ 0.01 ^†^
Corn	12	0.94 ± 0.0 ^b^	3.38 ± 0.0 ^b^
Wheat	12	0.95 ± 0.0 ^a^	3.50 ± 0.0 ^a^
Starch content		*p* ≤ 0.01 ^†^	*p* ≤ 0.01 ^†^
10%	8	0.94 ± 0.0 ^b^	3.25 ± 0.0 ^b^
15%	8	0.96 ± 0.0 ^a^	3.53 ± 0.0 ^a^
20%	8	0.95 ± 0.0 ^a^	3.53 ± 0.0 ^a^
3D Program		*p* = 0.12 ^‡^	*p* ≤ 0.01 ^†^
Program 1	12	0.95 ± 0.0 ^a^	3.35 ± 0.0 ^b^
Program 2	12	0.95 ± 0.0 ^a^	3.53 ± 0.0 ^a^
Dataset average	24	0.95 ± 0.0	3.53 ± 0.0

Results are expressed as mean ± standard error. Values represented with different letters are statistically different at *p* ≤ 0.05; ^†^ significant factor in multifactor analysis; ^‡^ not a significant factor in multifactor analysis. a_w_—activity of water (%).

**Table 3:** Association of 3DP processing parameters with the contents of bioactive compounds and antioxidant capacity in the 3DP samples.

Variable	*n*	TPC	HCA	FL	TF	ANT	CT	DPPH	FRAP
Starch type		*p* ≤ 0.01 ^†^	*p* ≤ 0.01 ^†^	*p* ≤ 0.01 ^†^	*p* ≤ 0.01 ^†^	*p* = 0.04 ^†^	*p* = 0.01 ^†^	*p* ≤ 0.01 ^†^	*p* ≤ 0.01 ^†^
Corn	12	74.47 ± 1.08 ^b^	19.44 ± 0.25 ^a^	6.90 ± 0.16 ^a^	5.30 ± 0.05 ^a^	7.50 ± 0.03 ^b^	43.90 ± 0.27 ^b^	487.83 ± 0.41 ^a^	1.19 ± 0.02 ^b^
Wheat	12	82.66 ± 1.08 ^a^	13.64 ± 0.25 ^b^	3.70 ± 0.16 ^b^	4.50 ± 0.05 ^b^	7.61 ± 0.03 ^a^	45.01 ± 0.27 ^a^	486.08 ± 0.41 ^b^	1.29 ± 0.02 ^a^
Starch content		*p* = 0.54 ^‡^	*p* = 0.09 ^‡^	*p* ≤ 0.01 ^†^	*p* = 0.04 ^†^	*p* ≤ 0.01 ^†^	*p* ≤ 0.01 ^†^	*p* ≤ 0.01 ^†^	*p* ≤ 0.01 ^†^
10%	8	77.4 ± 1.32 ^a^	16.00 ± 0.30 ^a^	4.47 ± 0.20 ^c^	4.86 ± 0.06 ^a,b^	7.82 ± 0.04 ^a^	45.02 ± 0.33 ^b^	515.76 ± 0.50 ^a^	1.24 ± 0.02 ^b^
15%	8	79.48 ± 1.32 ^a^	17.04 ± 0.30 ^a^	6.06 ± 0.20 ^a^	5.04 ± 0.06 ^a^	7.94 ± 0.04 ^a^	47.14 ± 0.33 ^a^	489.15 ± 0.50 ^b^	1.32 ± 0.02 ^a^
20%	8	78.83 ± 1.32 ^a^	16.58 ± 0.30 ^a^	5.37 ± 0.20 ^b^	4.80 ± 0.06 ^b^	6.91 ± 0.04 ^b^	41.20 ± 0.33 ^c^	455.97 ± 0.50 ^c^	1.16 ± 0.02 ^c^
3D Program		*p* = 0.03 ^†^	*p* = 0.08 ^‡^	*p* = 0.09 ^‡^	*p* ≤ 0.01 †	*p* ≤ 0.01 ^†^	*p* = 0.02 ^†^	*p* ≤ 0.01 ^†^	*p* = 0.08 ^‡^
Program 1	12	76.62 ± 1.08 ^b^	16.21 ± 0.25 ^a^	5.32 ± 0.16 ^a^	4.79 ± 0.05 ^b^	7.43 ± 0.03 ^b^	43.96 ± 0.27 ^b^	485.26 ± 0.41 ^b^	1.22 ± 0.02 ^a^
Program 2	12	80.51 ± 1.08 ^a^	16.87 ± 0.25 ^a^	5.28 ± 0.16 ^a^	5.01 ± 0.05 ^a^	7.69 ± 0.03 ^a^	44.95 ± 0.27 ^a^	488.66 ± 0.41 ^a^	1.27 ± 0.02 ^a^
Dataset average	24	78.57 ± 1.08	16.54 ± 0.18	5.30 ± 0.12	4.90 ± 0.04	7.56 ± 0.02	44.46 ± 0.19	486.96 ± 0.29	1.24 ± 0.01

Results are expressed as mean ± standard error. Values represented with different letters are statistically different at *p* ≤ 0.05; ^†^ significant factor in multifactor analysis; ^‡^ not a significant factor in multifactor analysis. TPC—Total phenolic compounds (mg 100 g^−1^); HCA—Hydroxycinnamic acids (mg 100 g^−1^); FL—Flavanols (mg 100 g^−1^); TF—Total flavonoids (mg 100 g^−1^); ANT—Monomeric anthocyanins (mg 100 g^−1^); CT—Condensed tannins (mg 100 g^−1^); DPPH assay (µM); FRAP assay (mM).

**Table 4:** The CIEL*a*b* color parameters in control and 3DP samples.

3DP Program	Type of Starch	Starch Content (%)	L*	a*	b*	C*	h (°)	ΔE*
Control sample	/	/	24.84	30.42	20.88	36.90	34.46	/
Program 1	corn	10	34.16	22.64	7.50	23.85	18.33	18.07
	corn	15	35.90	24.12	10.82	26.44	24.16	16.22
	corn	20	44.10	27.32	12.22	29.93	24.09	21.34
	wheat	10	38.64	27.52	11.76	29.93	23.14	16.79
	wheat	15	34.44	22.64	9.50	24.55	22.76	16.78
	wheat	20	39.54	24.46	9.74	26.33	21.71	19.38
Program 2	corn	10	34.20	23.10	8.86	24.74	20.98	16.91
	corn	15	33.52	22.54	10.04	24.67	24.01	15.97
	corn	20	43.94	26.68	12.98	29.67	25.94	21.01
	wheat	10	34.90	27.52	11.76	29.93	23.14	13.88
	wheat	15	33.40	22.8	11.10	25.36	25.96	15.07
	wheat	20	39.24	23.22	10.14	25.34	23.59	19.35

Control sample—sample without added starch; L*—lightness; a*—redness; b*—yellowness; ΔE*—color difference; C*—chroma; h*—hue angle.

**Table 5:** Influence of 3DP processing parameters on color, texture, particle diameters, and product dimensions expressed by *p*-value *.

Parameter	3DP Program	Type of Starch	Starch Content
L*	0.34	0.47	≤0.01 *
a*	0.72	0.82	0.29
b*	0.60	0.80	0.59
C*	0.89	0.82	0.42
h	0.17	0.66	0.11
Fp	0.07	0.03 *	0.04 *
Wp	0.11	0.26	0.02 *
F	1.00	≤0.01 *	0.02 *
W	0.99	≤0.01 *	0.02 *
D (3.2)	0.95	0.05 *	≤0.01 *
D (4.3)	0.95	0.05 *	≤0.01 *
d (0.1)	0.82	≤0.01 *	≤0.01 *
d (0.5)	0.85	0.85	≤0.01 *
d (0.9)	0.25	0.20	≤0.01 *
Length	0.51	0.06	0.23
Width	0.52	0.54	0.40
Height	0.76	0.78	0.16

L*—lightness; a*—redness; b*—yellowness; C*—chroma; h*—hue angle; F—extrusion force (firmness) (N); W—work at extrusion force (Nmm); F_p_ –maximum force (hardness) (N); W_p_—work at maximum force (Nmm); D (3.2)—surface weighted mean diameter (Sauter mean diameter); D (4.3)—volume weighted mean diameter (De Brouckere mean diameter); d (0.1)—10% of the volume distribution is below the observed diameter; d (0.5)—median diameter, 50% of the volume distribution is below, and 50% is above the observed diameter; d (0.9)—90% of the volume distribution is below the observed diameter. *—statistically significant (*p* < 0.05).

**Table 6:** Influence of 3DP processing parameters on the dimension of 3DP samples.

3DP Program	Type of Starch	Starch Content (%)	Length (mm)	Width (mm)	Height (mm)
Program 1	corn	10	53.36 ± 0.53	51.22 ± 0.49	12.24 ± 0.29
	corn	15	52.11 ± 0.56	51.45 ± 0.25	12.41 ± 0.23
	corn	20	52.12 ± 0.66	51.76 ± 0.31	12.49 ± 0.38
	wheat	10	52.25 ± 0.31	51.33 ± 0.51	11.72 ± 0.76
	wheat	15	52.26 ± 0.41	51.36 ± 0.14	12.25 ± 0.38
	wheat	20	51.76 ± 0.38	50.98 ± 0.91	12.36 ± 0.88
Program 2	corn	10	52.12 ± 0.89	51.11 ± 0.75	11.87 ± 0.48
	corn	15	53.28 ± 0.39	51.25 ± 0.57	11.81 ± 0.71
	corn	20	52.23 ± 0.62	51.34 ± 0.67	12.66 ± 0.43
	wheat	10	52.25 ± 0.54	51.25 ± 0.55	12.41 ± 0.27
	wheat	15	51.56 ± 0.37	51.46 ± 0.77	12.49 ± 0.57
	wheat	20	51.22 ± 0.67	52.91 ± 0.39	12.55 ± 0.64

Results are presented as an average value of triplicate measurements ± STDEV.

**Table 7:** Influence of 3DP parameters on textural properties of samples.

3DP Program	Type of Starch	Starch Content (%)	F (N)	W (Nmm)	F_p_ (N)	W_p_ (Nmm)
Program 1	corn	10	53.538	535.200	0.143	0.115
	corn	15	50.437	504.279	0.278	0.116
	corn	20	95.881	958.52	0.18	0.129
	wheat	10	98.543	985.129	0.073	0.074
	wheat	15	177.914	1778.729	0.147	0.149
	wheat	20	162.755	1627.201	0.139	0.145
Program 2	corn	10	39.367	393.599	0.084	0.077
	corn	15	37.723	377.149	0.14	0.092
	corn	20	92.904	917.987	0.173	0.106
	wheat	10	99.029	990.085	0.085	0.081
	wheat	15	182.785	1827.473	0.126	0.118
	wheat	20	187.259	1872.168	0.108	0.143

F—extrusion force (firmness) (N); W—work at extrusion force (Nmm); F_p_—maximum force (hardness) (N); W_p_—work at maximum force (Nmm).

**Table 8:** Influence of 3DP processing parameters on particle diameters of 3DP samples.

3DP Program	Type of Starch	Starch Content (%)	D (3.2) (µm)	D (4.3) (µm)	d (0.1) (µm)	d (0.5) (µm)	d (0.9) (µm)
Program 1	corn	10	49.56	23.563	21.391	122.724	598.039
	corn	15	25.763	116.602	12.901	35.68	357.256
	corn	20	22.458	76.682	11.468	31.748	234.055
	wheat	10	44.827	176.767	22.855	74.155	489.584
	wheat	15	30.226	90.956	15.759	46.31	168.747
	wheat	20	27.99	94.021	14	43.511	287.481
Program 2	corn	10	44.133	221.151	19.313	95.741	591.564
	corn	15	25.945	119.374	12.852	36.627	364.902
	corn	20	21.959	84.936	10.757	31.725	262.309
	wheat	10	48.053	201.603	24.092	84.512	541.467
	wheat	15	33.328	126.287	16.924	51.738	362.298
	wheat	20	28.011	92.548	13.804	43.546	270.289

D (3.2)—surface weighted mean diameter (Sauter mean diameter); D (4.3)—volume weighted mean diameter (De Brouckere mean diameter); d (0.1)—10% of the volume distribution is below the observed diameter; d (0.5)—median diameter, 50% of the volume distribution is below and 50% is above the observed diameter; d (0.9)—90% of the volume distribution is below the observed diameter.

**Table 9:** Microbiological counts (CFU g^−1^) of the 3DP samples during 10 days of storage at 4 °C.

Microorganism Type	Sample	Days of Storage				
		0	2	4	7	10
Aerobic mesophilic bacteria	control	1.5 × 10^2^	9 × 10^2^	1.8 × 10^3^	n.d.	n.d.
	vanillin 1 g L^−1^	n.d.	9 × 10^2^	9 × 10^2^	3.8 × 10^4^ *	9 × 10^4^ *
	vanillin 2 g L^−1^	n.d.	n.d.	9 × 10^4^ *	n.d.	9 × 10^2^
	citral 75 mg L^−1^	1.8 × 10^3^	n.d.	n.d.	n.d.	n.d.
	citral 150 mg L^−1^	1.5 × 10^2^	9 × 10^2^	3,6 × 10^3^	n.d.	n.d.
*Enterobacteriaceae*	control	n.d.	n.d.	n.d.	n.d.	n.d.
	vanillin 1 g L^−1^	n.d.	n.d.	n.d.	n.d.	n.d.
	vanillin 2 g L^−1^	n.d.	n.d.	n.d.	n.d.	n.d.
	citral 75 mg L^−1^	n.d.	n.d.	n.d.	n.d.	n.d.
	citral 150 mg L^−1^	n.d.	n.d.	n.d.	n.d.	n.d.
*Salmonella* sp.	control	n.d.	n.d.	n.d.	n.d.	n.d.
	vanillin 1 g L^−1^	n.d.	n.d.	n.d.	n.d.	n.d.
	vanillin 2 g L^−1^	n.d.	n.d.	n.d.	n.d.	n.d.
	citral 75 mg L^−1^	n.d.	n.d.	n.d.	n.d.	n.d.
	citral 150 mg L^−1^	n.d.	n.d.	n.d.	n.d.	n.d.
*Escherichia coli*	control	n.d.	n.d.	n.d.	n.d.	n.d.
	vanillin 1 g L^−1^	n.d.	n.d.	n.d.	n.d.	n.d.
	vanillin 2 g L^−1^	n.d.	n.d.	n.d.	n.d.	n.d.
	citral 75 mg L^−1^	n.d.	n.d.	n.d.	n.d.	n.d.
	citral 150 mg L^−1^	n.d.	n.d.	n.d.	n.d.	n.d.
Yeasts and molds	control	n.d.	n.d.	n.d.	n.d.	n.d.
	vanillin 1 g L^−1^	n.d.	n.d.	n.d.	n.d.	n.d.
	vanillin 2 g L^−1^	n.d.	n.d.	n.d.	n.d.	n.d.
	citral 75 mg L^−1^	n.d.	n.d.	n.d.	n.d.	n.d.
	citral 150 mg L^−1^	n.d.	n.d.	n.d.	n.d.	n.d.

Control—sample without antimicrobial agents; vanillin 1 g L^−1^—3DP sample with addition of vanillin in a concentration of 1 g L^−1^; vanillin 2 g L^−1^—3DP sample with addition of vanillin in a concentration of 2 g L^−1^; citral 75 mg L^−1^—3DP sample with addition of citral in a concentration of 75 mg L^−1^; citral 150 mg L^−1^—3DP sample with addition of citral in a concentration of 150 mg L^−1^; n.d.—not detected; above the safety limit of 10^4^ CFU g^−1^; *—not satisfactory criterion (≤10^4^ CFU mL^−1^).

## Data Availability

Not applicable.
